# Kinetic regulation of the binding of prothrombin to phospholipid membranes

**DOI:** 10.1007/s11010-013-1735-2

**Published:** 2013-06-28

**Authors:** Emma Smith, Rina Vekaria, Katherine A. Brown, Colin Longstaff

**Affiliations:** 1 Division of Cardiovascular and Diabetes Research, The LIGHT Laboratories, University of Leeds, Leeds, UK; 2 PPSI, Uxbridge, Middlesex, UB8 1LZ UK; 3 Institute of Cellular and Molecular Biology, The University of Texas at Austin, Austin, TX 78712 USA; 4 Cavendish Laboratory, University of Cambridge, Cambridge, CB3 0HE UK; 5 Biotherapeutics Section, National Institute for Biological Standards and Control, South Mimms, Hertfordshire EN6 3QG UK

**Keywords:** Membranes, Phospholipid binding, Prothrombin, Blood coagulation, Surface plasmon resonance

## Abstract

A wide range of equilibrium and kinetic constants exist for the interaction of prothrombin and other coagulation factors with various model membranes from a variety of techniques. We have investigated the interaction of prothrombin with pure dioleoylphosphatidylcholine (DOPC) membranes and dioleoylphosphatidlyserine (DOPS)-containing membranes (DOPC:DOPS, 3:1) using surface plasmon resonance (SPR, with four different model membrane presentations) in addition to isotheral titration calorimetry (ITC, with suspensions of phospholipid vesicles) and ELISA methods. Using ITC, we found a simple low-affinity interaction with DOPC:DOPS membranes with a *K*_D_ = 5.1 μM. However, ELISA methods using phospholipid bound to microtitre plates indicated a complex interaction with both DOPC:DOPS and DOPC membranes with *K*_D_ values of 20 and 58 nM, respectively. An explanation for these discrepant results was developed from SPR studies. Using SPR with low levels of immobilised DOPC:DOPS, a high-affinity interaction with a *K*_D_ of 18 nM was obtained. However, as phospholipid and prothrombin concentrations were increased, two distinct interactions could be discerned: (i) a kinetically slow, high-affinity interaction with *K*_D_ in the 10^−8^ M range and (ii) a kinetically rapid, low-affinity interaction with *K*_D_ in the 10^−6 ^M range. This low affinity, rapidly equilibrating, interaction dominated in the presence of DOPS. Detailed SPR studies supported a heterogeneous binding model in agreement with ELISA data. The binding of prothrombin with phospholipid membranes is complex and the techniques used to measure binding will report *K*_D_ values reflecting the mixture of complexes detected. Existing data suggest that the weaker rapid interaction between prothrombin and membranes is the most important in vivo when considering the activation of prothrombin at the cell surface.

## Introduction

It is generally accepted that assembly of blood coagulation factor complexes and initiation of coagulation requires a phospholipid membrane surface [1, 2]. The role of the phospholipid surface has been explained in a number of ways: (i) enhancing the local concentrations of reactants; (ii) inducing conformational changes and promoting optimal alignment; (iii) enhancing substrate delivery and product removal; and (iv) restricting coagulation to areas of injury (reviewed [3]). However, the mechanism of binding of coagulation factors to model phospholipid membranes, let alone heterogeneous cell membranes [4], is not completely understood [5]. The vitamin K-dependent family of membrane-binding proteins (prothrombin, and Factors VII, IX, X, XI) involved in coagulation display γ-carboxyglutamic acid (Gla) residues in a highly conserved N-terminal domain [6]. Negatively charged Gla residues bind Ca^2+^ ions which both maintain a conformation with an exposed hydrophobic ω-loop ready for membrane insertion, and bridge Gla domains with anionic phospholipid, primarily phosphatidylserine (PS), which is exposed on the external leaflet of cell membranes following damage. In vivo, the importance of PS exposure in maintaining haemostasis is well recognised (reviewed [7]), and when the scrambling of PS from inner to outer cell membrane is defective, bleeding abnormalities can occur [8]. However, the precise structural details of the bound clotting factor optimised for coagulation are still debated and a complete understanding of coagulation factor–membrane binding has been further complicated by the wide range of results obtained for equilibrium constants published over many years in studies using a variety of techniques with model membranes. The importance of the kinetics of binding and enzyme activation has also been highlighted in a number of studies (e.g. [9]).

A number of methods are available to study the binding of peripheral membrane proteins, such as prothrombin, to model membranes. In the present work, the interactions of prothrombin with phospholipid were studied using a range of complementary equilibrium and kinetic techniques, where phospholipids are presented in a variety of common formats, which may be more or less analogous to natural biological membranes. Isothermal titration calorimetry (ITC) requires no labelling of reactants and works on soluble proteins and phospholipid suspensions, but is relatively insensitive, requiring high concentrations of protein and well suited for studying equilibrium constants in the 10^−7^–10^−8^ M range. ELISA is a sensitive technique requiring only low concentrations of proteins, but useful only for high-affinity interactions. ELISA also involves manipulation and immobilisation of phospholipid and several processing steps. The optical phenomenon of surface plasmon resonance (SPR) offers several advantages over previous methods, since it does not require labelling of the reactants, wide ranges of ligands and analyte concentrations can be studied, and the kinetics of binding are observed directly in real time allowing determination of rate constants in a straightforward manner [10]. Furthermore, a number of different model membrane systems are available which minimises the risk of model-specific artefacts. We have used SPR to investigate prothrombin binding to phospholipid presented in four forms, including a supported monolayer prepared on an alkanethiol hydrophobic surface (HPA Sensor Chips), immobilised phospholipid prepared on a dextran surface via hydrophobic linkers (L1 Sensor Chips), phospholipid vesicles tethered via incorporated biotinylated phosphatidylethanolamine (PE) and immobilised by an anti-biotin antibody chemically coupled to either dextran (dextran coated CM5 and CM4 sensor chips), or to surface carboxyl groups (C1 sensor chips). The results from kinetic studies using SPR have been used to develop a model for prothrombin binding to membranes that rationalises varying results obtained in different systems and aids understanding of the mechanisms of prothrombin activation in vivo. These approaches are also relevant to other studies of protein–membrane interactions. Nevertheless, it should be emphasised that protein–membrane interactions are only one component regulating coagulation reactions that lead to thrombin generation. Binding and processing of prothrombin by membrane–factor Va–factor Xa (‘prothrombinase’), for example, as occur in vivo will inevitably involve more complexities (e.g. [11]) than will be seen in systems composed of purified model membranes.

## Materials and methods

### Materials

The synthetic lipids 1,2-Dioleoyl-*sn*-glycerol-3-phosphocholine (DOPC) and 1,2-Dioleoyl-*sn*-glycerol-3-phospho-l-serine, sodium salt (DOPS) were purchased from Alexis Corporation Ltd (Nottingham, UK) or from Avanti Polar Lipids (Alabaster, USA). Biotinylated 1,2-Dioleoyl-*sn*-glycero-3-phosphoethanolamine (DOPE) was supplied by Avanti Polar Lipids and polyclonal rabbit anti-biotin IgG was from Rockland Immunochemicals for Research (Gilbertsville, USA). Buffers used in these studies were buffer A (10 mM HEPES, 150 mM NaCl, 5 mM CaCl_2_, pH 7.4) for experiments performed in the presence of Ca^2+^ ions, and buffer B (10 mM HEPES, 150 mM NaCl, 3.4 mM EDTA, pH 7.4) for experiments performed in the absence of Ca^2+^ ions. Prothrombin (human and bovine) was purchased from Enzyme Research Laboratories (Swansea, UK) and was dialysed into buffer either A or B before use. Prothrombin concentration was determined by measuring the absorbance at 280 nm using *E* ^1 %^ = 13.6 and molecular mass = 72 kDa. No contaminating proteins were detected in SDS-PAGE analysis. *N*-octyl-ß-d-glucopyranoside (*n*-octylglucoside) was purchased from Calbiochem Novabiochem Ltd (Nottingham, UK). Human serum albumin (HSA) used was a 20 % (w/v) solution from Immuno Ltd (Vienna, Austria).

### Preparation and immobilisation of phospholipid

In all the cases, vesicles were prepared prior to formation of the phospholipid surface at a final concentration of 2 mg ml^−1^ as pure DOPC or DOPC:DOPS, 3:1 (25 % DOPS). DOPC and DOPS stock solutions of 50 and 10 mg ml^−1^, respectively, were prepared in chloroform:methanol (1:1) and aliquots stored in liquid nitrogen vapour phase. Aliquots of DOPC and DOPC:DOPS in the appropriate ratios were dried down under a stream of nitrogen gas and resuspended in buffer A or B, with or without 5 mM Ca^2+^ as appropriate. These phospholipid suspensions were sonicated on ice/water for 5 × 30 s and filtered using a 0.2-μm filter (Sartorius AG, Göttingen, Germany), then extruded through a 50-nm filter (LipoFast Extruder, Milsch Equipment Laudenbach, Germany) to create small, unilamellar vesicles. Phospholipid vesicles tethered via anti-biotin to CM5, CM4 and C1 sensor chips were prepared similarly except for the inclusion of 2 % (w/v) biotinylated DOPE.

### ITC

ITC experiments were performed by Dr Alan Cooper (Department of Chemistry, University of Glasgow, Glasgow, Scotland). Experiments to measure the binding of prothrombin to DOPC or DOPC:DOPS vesicles were performed at 25 °C using a Microcal OMEGA titration microcalorimeter following standard instrumental procedures (Cooper and Johnson, 1994; Wiseman et al., 1989) with a 250-μl injection syringe and 400 rpm stirring. Samples were prepared by rigorous dialysis in buffer A and degassed gently, immediately before use. Prothrombin concentration in the ITC injection syringe was 0.15 mM, and phospholipid concentration (in the ITC cell) was 2.5 mM. A typical binding experiment involved 25 × 10 μl injections of prothrombin solution into the ITC cell (1.3 ml active volume) containing vesicles. Control experiments were performed under identical conditions by injection of protein into buffer alone (to correct for heats of protein dilution). Corrected data were plotted as a binding isotherm as the total heat energy per unit time (μcal s^−1^) from each injection as determined from the area underneath the injection peak against the molar ratio of ligand to macromolecule. The curve was fitted by nonlinear regression using a simple single-site-binding model in the Microcal ORIGIN software package (Microcal, Northampton, USA). For each thermal titration curve, this yields estimates of the binding stoichiometry (*N*), the binding constant (*K*_A_/M^−1^) and the change in enthalpy of binding (Δ*H*/kcal mol^−1^). The energetics of binding are expressed by the Gibbs free energy of binding (Δ*G*).

### ELISA

Two methods for immobilising phospholipid to microtitre plates were used. In method 1, suspensions of 25 μg ml^−1^ phospholipids and 100 μl volumes of chrloroform:methanol (1:1) were prepared and dried directly onto the surface of the well under a stream of nitrogen gas for 1 h at 37 °C. Wells were then blocked with either buffer A or B, as appropriate, containing 1 % (w/v) casein. Prothrombin dilution ranges were made in either buffer A or B, as appropriate, containing 1 mg ml^−1^ HSA and incubated with immobilised phospholipid for 1 h before washing and incubation with 5 μg ml^−1^ of goat anti-human prothrombin for a further hour. In method 2, suspensions of 100 μM phospholipids were prepared as described for ITC and SPR work, and then incubated in microtiter plates for 1 h at 37 °C. Wells were blocked and prothrombin incubated as described above for method 1. In both methods, detection of bound antibodies was quantified using 0.1 μg ml^−1^ peroxidase-conjugated rabbit anti-goat immunoglobulin.

### SPR

The SPR device used for the binding studies was the Biacore-X or Biacore 3000 instrument, and all the sensor chips were purchased from Biacore AB (Uppsala, Sweden). Sensor chips were treated in accordance with the manufacturer’s instructions before injection of phospholipid. Complete coverage of HPA surfaces was assessed by injection of 10 μl of 1.5 μM HSA at a flow rate of 10 μl min^−1^ and a fully coated surface typically bound less than 30 resonance units (RU) after a 1 min injection. Vesicles were tethered to CM5, CM4 or C1 surfaces via a polyclonal rabbit anti-biotin antibody which was coupled to the chip using standard NHS–EDC techniques [12]. Kinetic studies were carried out using the prothrombin concentrations given in Results using a flow rate of 20 μl min^−1^. Regeneration of the HPA surface was successfully achieved with 10 mM NaOH to remove the bound protein from the stable phospholipid monolayer or with sequential injections of 25 mM HCl and 2 % (v/v) *n*-octylglucoside detergent in the case of CM5, CM4 or C1 surfaces, or 50 mM NaOH and 2 % (v/v) *n*-octylglucoside for L1 sensor chip surfaces to remove both phospholipid and protein.

Biomolecular interactions on the sensor chip surface are monitored as changes in RU recorded as a sensorgram, a plot of RU against time. Data from a reference channel of immobilised antibody, no phospholipid, was subtracted from the active phospholipid-bound channel where possible (CM5, CM4 and C1 surfaces). Sensorgrams were routinely analysed using the BIAevaluation 4.1 package [13] by global curve fitting to a complete set of curves over a range of prothrombin concentrations, to derive values for association rate constants (*k*_a_) and dissociation rate constants (*k*_d_) and maximum binding (*R*_max_). Initially, a simple 1:1 Langmuir binding model was investigated, then more complex models as required and selected using evidence from other techniques.

## Results

### ITC

**Fig. 1:**
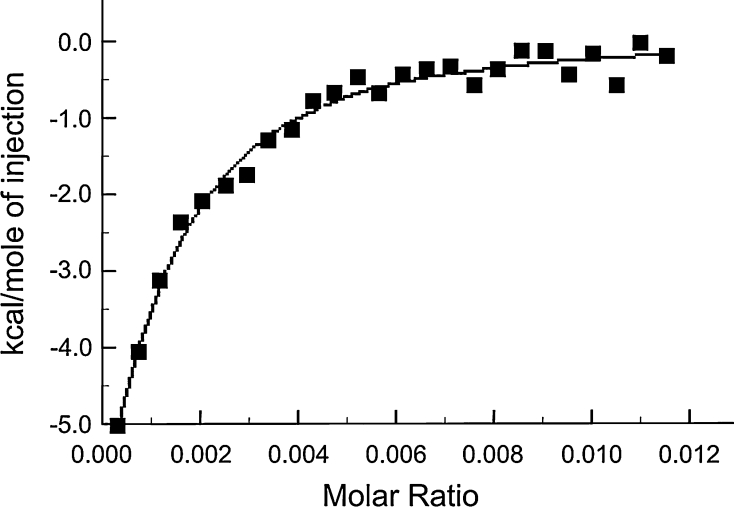
Binding of prothrombin to a suspension of phospholipid vesicles of DOPC:DOPS, 3:1, in the presence of 5 mM Ca^2+^ at 25 °C assessed by isothermal titration calorimetry (ITC). The binding isotherm is shown fitted to data collected for heat generated from 25 × 10 μl injections of prothrombin solution into the ITC cell. The equilibrium constant, *K*_A_, from the fitting was (19.7 ± 3.8) × 10^4^ M^−1^, giving a *K*_D_ value of 5.1 μM. The change in enthalpy, Δ*H*, was (19.56 ± 9.26) kcal mol^−1^, and the calculated Δ*G* value was −7.22 kcal mol^−1^

ITC is commonly used to obtain thermodynamic data and measure binding affinities for macromolecular interactions. Affinities are calculated from the heat absorbed or released during a binding reaction, and there is no need for labelling or immobilisation of the reactants. However, high concentrations of samples are required to observe the heat changes. In the results shown in Fig. 1 for the binding of prothrombin to a suspension of phospholipid vesicles of DOPC:DOPS, 3:1, protein concentrations up to 0.150 μM were required to ensure a significant signal. However, good data were obtained in the presence of 5 mM Ca^2+^ ions. The equilibrium constant, *K*_A_, from the fitting was (19.7 ± 3.8) × 10^4^ M^−1^, giving a *K*_D_ value of 5.1 μM. The change in enthalpy, Δ*H*, was −(19.6 ± 9.3) kcal mol^−1^ and the calculated Δ*G* value was −7.22 kcal mol^−1^, indicating that the formation of prothrombin–phospholipid complexes is favourable. Binding to DOPC vesicles could not be studied as the signals obtained were too weak and no binding was observed in the absence of 5 mM Ca^2+^ ions.

### ELISA studies

**Fig. 2:**
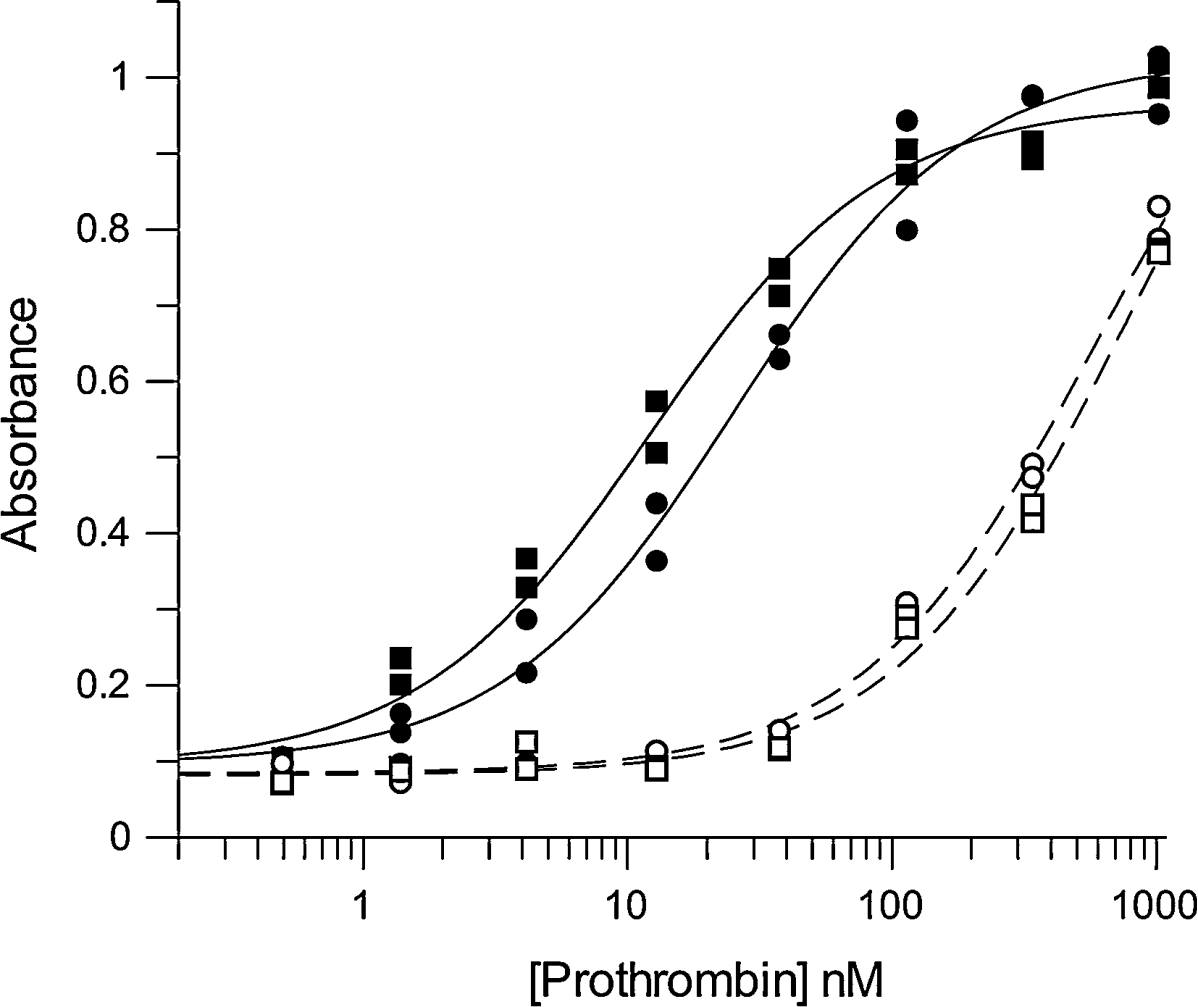
Representative data for ELISA detection of prothrombin bound to microtitre plates coated with phospholipid. A range of prothrombin concentrations were incubated with phospholipid coated onto microtitre plate wells, washed and bound prothormbin detected using anti-prothrombin antibodies. Data are shown for replicate experiments in the presence (*solid symbols*) or absence (*open symbols*) of 5 mM Ca^2+^. The phospholipid surfaces used were DOPC (*circles*) or DOPC;DOPS, 3:1 (*squares*). Single-site-binding isotherms were fitted and are shown as *solid lines* or *broken lines* in the presence or absence of Ca^2+^, respectively

Binding of prothrombin to phospholipid was also studied using ELISA-based methodology, where phospholipid was immobilised to microtitre plates by incubation of a vesicle suspension or by drying phospholipid onto the plate surface from chloroform:methanol (1:1) under a stream of nitrogen. Detection of bound prothrombin was the same for both the methods and a typical example of results from such an experiment is shown in Fig. 2 for phospholipid immobilised by incubation with microtitre plates. In this case, there was consistently better binding to DOPC:DOPS, but nevertheless some observable binding to immobilised DOPC. There was very low background binding over this range of prothrombin in the absence of Ca^2+^ ions, but some binding was evident at the highest prothrombin concentrations. Combined results from 12 independent experiments resulted in apparent *K*_D_ values for DOPC:DOPS, 3:1, and DOPC surfaces of 20.3 ± 9.5 and 57.7 ± 27.1 nM, respectively (mean ± SD). A paired *t* test indicated that these two *K*_D_ values were significantly different at the 0.05 % level. Data from plates where phospholipid was dried onto the surface from solvent tended to give slightly higher affinities. Scatchard transformations of binding isotherms were always nonlinear, suggesting some complexity in the binding mechanism; therefore, the *K*_D_ values determined represent a simple overall approximation of binding affinity. Given the small difference in *K*_D_ values for DOPC and DOPC:DOPS surfaces with prothrombin, and the tendency for nonspecific binding in the absence of Ca^2+^ ions at high-prothrombin concentrations, it is possible that binding is not purely dominated by phospholipid–Gla domain interactions in these ELISA systems.

### Prothrombin interaction with model membranes studied by SPR

**Fig. 3:**
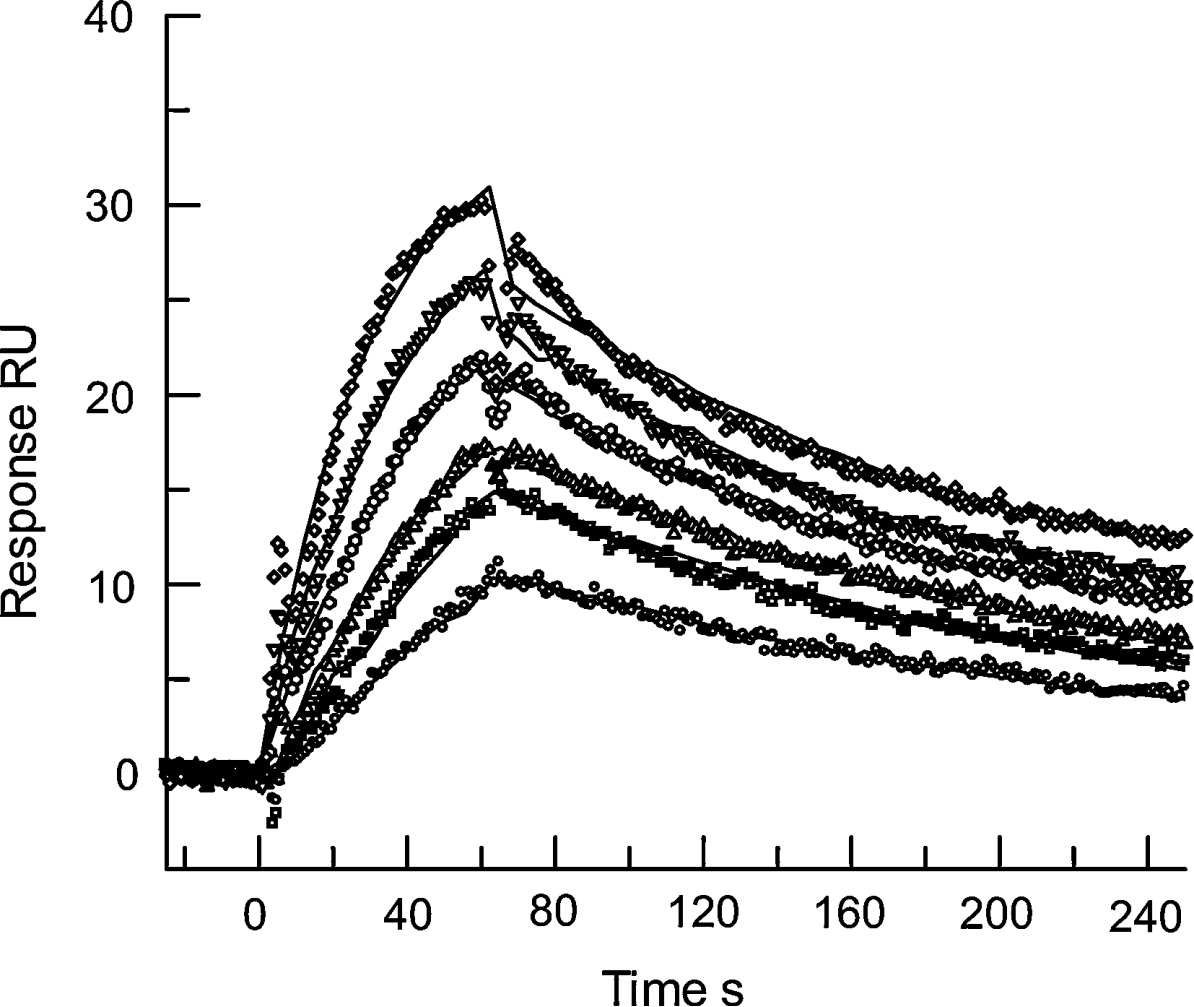
Sensorgrams showing binding kinetic of prothrombin binding to low levels of vesicles of DOPC:DOPS, 3:1, tethered to a CM5 sensor chip in the presence of 5 mM Ca^2+^. The surface was prepared by immobilising approximately 5 ng of anti-biotin antibody which was able to capture 70 RU of phospholipid vesicles-containing 2 % biotinylated DOPE. Immobilisation of phospholipid vesicles was followed by injection of a 50–200 nM prothrombin and the maximum capacity of prothrombin binding to this surface was approximately 35 RU. The surface was regenerated after each prothrombin dissociation by sequential injections of 25 mM HCl and 2 % (v/v) *n*-octylglucoside, which removed phospholipid and bound prothrombin, before injection of fresh phospholipid. The *solid lines* show global, simultaneous fitting of sensorgrams using a simple 1:1 Langmuir binding model. Kinetic parameters were *k*_a_ = (2.75 ± 0.03) × 10^5^ M^−1^ s^−1^, *k*_d_ = (4.98 ± 0.05) × 10^−3^ s^−1^ and *K*_D_ = 18.1 ± 0.3 nM

A number of different phospholipid surfaces were investigated by SPR. The most detailed studies involved vesicles tethered through incorporated biotinylated DOPE to anti-biotin antibodies chemically immobilised to the sensor chip surface. These surfaces permit subtraction of nonspecific-binding data from a reference channel and allow control of the amount of phospholipid immobilised. Furthermore, regeneration is optimised as the tethered vesicles and membrane-bound proteins can be completely removed from the sensor chip using detergent and acid treatment, so that each subsequent injection of prothrombin is on a fresh phospholipid surface. Detailed kinetic investigations were performed on a CM5 sensor chip surface with a low level of immobilised phospholipid, as shown in Fig. 3. This surface was prepared by immobilising approximately 5 ng of anti-biotin antibody which we found was able to capture 70 RU of DOPC:DOPS vesicles containing 2 % biotinylated DOPE. Immobilisation of phospholipid vesicles was followed by injection of 50–200 nM prothrombin so that kinetics of binding and dissociation could be followed. The maximum capacity of prothrombin binding to this surface was approximately 35 RU. In this case, global, simultaneous fitting of sensorgrams was successful using a simple 1:1 Langmuir binding model. Kinetic parameters were *k*_a_ = (2.75 ± 0.03) × 10^5^ M^−1^ s^−1^, *k*_d_ = (4.98 ± 0.05) × 10^−3^ s^−1^ and *K*_D_ = 18.1 ± 0.3 nM. Data and fittings are shown in Fig. 3. However, closer inspection focusing on the dissociation phases indicated that two dissociation curves were present that could be better fitted to a double exponential decay (not shown). Fitting was not significantly improved using global fitting to complex heterogeneous or two-step models because at these low concentrations sparse data are available from the faster kinetic dissociation phase. There was little or no specific binding of prothrombin to immobilised DOPC at this level of phospholipid on this surface and no kinetic or affinity constants could be derived.

At higher concentrations of prothrombin, some nonspecific binding to the dextran surface of the CM5 chip was observed which was eliminated using a CM4 chip surface, presumably as a result of less nonspecific binding on the less highly charged CM4 surface. Further detailed studies using 2 ng anti-biotin antibody immobilised onto a CM4 chip capturing vesicles-containing biotinylated PE, were performed to investigate the injection of 500 nM prothrombin for various times from 30 s to 8 min before dissociation was monitored. The mean maximum level of phospholipid immobilised over this series of injections was 94.9 ± 2.8 RU (mean ± SD), and saturation of phospholipid binding sites was reached after a 1 min injection of 500 nM prothrombin achieving 35.9 ± 0.9 RU of bound prothrombin. Again, close examination of the dissociation curves indicated double exponential decay model fitted better, but the rate constant for the rapid phase could not be reliably determined due to limited amount of data available and the fast rate. Significantly, there was no change in the pattern of the dissociation curves over increasing injection time from 30 s to 8 min (data not shown) indicating that prothrombin binds to heterogeneous-binding sites on DOPC:DOPS vesicles. If the dissociation rate constant had decreased, this would have suggested a two-step reaction mechanism where a stable complex accumulated over time [14].

**Fig. 4:**
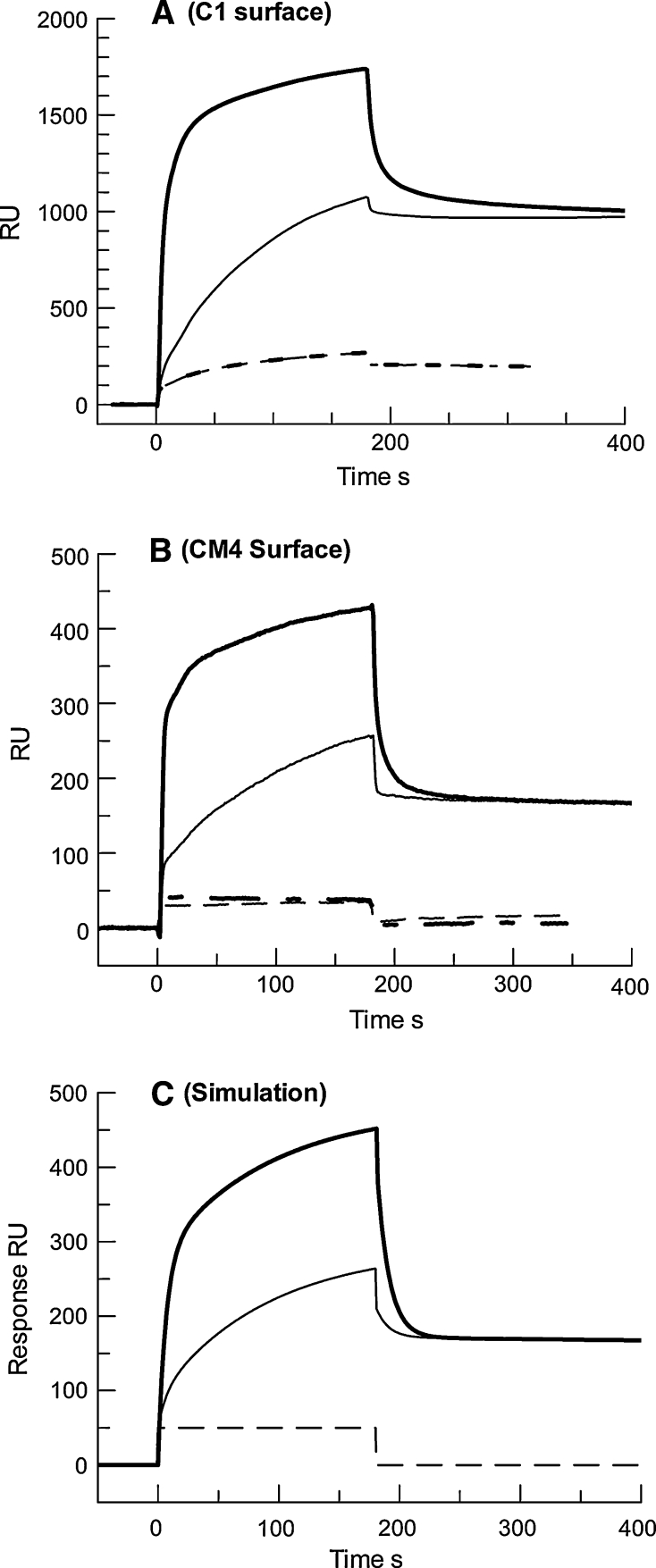
Binding of prothrombin to high levels of vesicles of DOPC or DOPC:DOPS, 3:1, tethered to a C1 sensor chip or a CM4 sensor chip. **a** shows overlaid sensorgrams for 1,100 nM prothrombin binding to 2,200 RU of phospholipid vesicles-containing 2 % biotinylated DOPE. **b** shows sensorgrams for binding of 1,100 nM prothrombin to 1,200 RU of the same phospholipid vesicles tethered to a CM4 sensor chip. The *heavy lines* show binding to the DOPC:DOPS, 3:1, surface and the *thinner lines* show binding to the DOPC surface. *Solid lines* and *dashed lines* show data in the presence and absence of 5 mM Ca^2+^, respectively. **c** shows simulated sensorgrams for the same conditions generated by the BIAevaluation software used to fit kinetic data. The *heavy solid line* is for the binding of 1,100 nM prothrombin following a heterogeneous-binding model with two components, *R*_max_ 1 = 650 RU, *k*_d_1 = 5 × 10^4^ M^−1^ s^−1^, *k*_d_1 = 0.1 s^−1^ and *K*_D_ = 2 μM; *R*_max_2 = 200 RU, *k*_a_2 = 1 × 10^4^ M^−1^ s^−1^, *k*_d_2 = 0.0001 s^−1^ and *K*_D_ = 10 nM. The *thin line* is simulated binding following the same kinetics, but *R*_max_1 is reduced to 120 RU. A bulk refractive index change of 50 RU is included in all sensorgrams and is shown as the *dashed line*

To investigate the faster dissociating complex in more detail, other phospholipid surfaces were prepared with higher levels of phospholipid immobilised and higher concentrations of prothrombin used. Phospholipid surfaces included CM4 (1,200 RU phospholipid) and C1 (2,200 RU phospholipid) with higher levels of anti-biotin antibody, an L1 sensor chip which immobilises a bilayer of phospholipid (3,000 RU phospholipid), and the HPA sensor chip that supports a phospholipid monolayer (1,300 RU phospholipid). All surfaces gave essentially the same patterns of binding, exemplified in Fig. 4a for a CM1 sensor chip charged with 2,200 RU phospholipid. Examination of the representative curves shown in Fig. 4a suggests that the DOPC surface bound prothrombin with slow kinetics, whereas the addition of DOPS to the phospholipid surface added an additional binding component with rapid on/off kinetics. It was not possible to adequately fit a two-site heterogeneous ligand model over a range of prothrombin concentrations (from 10 nM to 2 μM), indicating further complexity in the binding reactions. However, using this physiological concentration of prothrombin as shown in Fig. 4, it was relatively straightforward to replicate the curves in Fig. 4a and b, as shown in Fig. 4c using a heterogeneous ligand-binding model with rapid kinetics (*R*_max_1 = 650, *k*_a_1 = 5 × 10^4^ M^−1^ s^−1^, *k*_d_1 = 0.1 s^−1^ and *K*_D_ = 2 μM) and with slow kinetics (*R*_max_2 = 200, *k*_a_2 = 1 × 10^4^ M^−1^ s^−1^, *k*_d_2 = 1 × 10^−4^ s^−1^ and*K*_D_ = 10 nM). In the presence of DOPS, both reactions take place, but in the presence of PC only (*R*_max_1 has been reduced to 120) the kinetically rapid reaction is drastically reduced. A bulk refractive index signal of 50 RU was included in all the curves which accounts for most of the response seen with prothrombin in the absence of Ca^2+^ with either phospholipid surface as shown in Fig. 4.

## Discussion

### Prothrombin affinity for phospholipid

Previously published estimates for *K*_D_ for prothrombin binding to PC:PS vary over a wide range from high affinity ≤10^−8^ M range (e.g. [15–17]) to other values in the 10^−7^ M range or >10^−6^ M [18, 19]. Kinetic studies have also provided highly variable rate constants spanning many orders of magnitude [20, 21]. We can propose an explanation for this variability based on the results obtained in this study using different methods. Crucially, our results using SPR differentiate between two different types of interactions with phospholipid membranes: a rapid on–off reaction which is apparent in the presence of DOPS-containing membranes, and a slower reaction which takes place with model membranes composed of both DOPC and mixtures of DOPC:DOPS. The detection of complexes from these reactions will depend on the techniques used. For example, ELISA methods are sensitive and are able to detect high affinity, kinetically slow interactions, since long incubations are typically part of an ELISA protocol allowing slow reactions to reach equilibrium, while washing steps will remove loosely associated proteins with fast dissociation rates. On the other hand, ITC, which requires relatively high concentrations of prothrombin and phospholipid, identified a binding isotherm with a *K*_D_ value ≈ 5 μM and in line with the rapid on–off reaction observed in the SPR work. No binding to DOPC could be reliably detected in the absence of DOPS when using ITC.

### Kinetics, mechanism and structures

SPR studies have the advantage of being able to measure the binding over a wide range of affinities (*K*_D_ of the order 10^−9^–10^−4^ M) and kinetic rate constants, and are thus useful for exploring mechanisms of binding [10]. Both SPR and ELISA data (Scatchard plots) discussed above support a complex mechanism of binding where binding sites with a range of *K*_D_ values are present. Different types of interaction such as ionic bonding involving bridging Ca^2+^ ions, hydrophobic ω-loop insertion into the membrane and direct interactions between DOPS and prothrombin amino acid side chains [5] could all account for the heterogeneity of binding observed. Other complicating factors are also possible and not considered in our basic heterogeneous model, including association of protein molecules before and after the membrane binding e.g. [15, 22, 23], and possible re-ordering of the phospholipid around associating protein [16].

The nature and curvature of the phospholipid surface may also affect the kinetics and equilibrium constants determined and explain some variations seen among the different SPR methods with immobilised vesicles (on CM5, CM4 and C1 sensor chips) or planar membranes (HPA monolayer or L1 bilayer). A SPR study on coagulation factor binding to vesicles tethered using cholesterol attached to DNA tags concluded that membrane curvature was important for kinetics and amount of binding, but less so for the overall equilibrium constant (changes in association and dissociation tended to cancel out) [17]. Interestingly, in this study [17], prothrombin-binding kinetics were complicated and not analysed fully, but the *K*_D_ value with the DNA-tethered vesicles was estimated to be 68 nM, close to our tight-binding complex. Erb et al. [24] have also studied Gla-dependent coagulation factor X (FX) binding to phospholipid membranes by SPR. Again a complex binding mechanism was observed with multiple dissociation phases, indicating multiple steps, and also some binding to pure PC membranes was reported. Interestingly, isolated Gla domains bound with a *K*_D_ value around 5 μM and full length FX with a *K*_D_ ≈ 40 nM, again suggesting high-affinity binding is mediated by multiple binding sites. Recent SPR studies using a L1 DOPC:DOPS surface found complex binding and dissociation kinetics and favoured a two-step mechanism arising from conformation changes in prothrombin following membrane binding noted in previous studies [25]. However, this is not in accord with our SPR studies using long injections which support the heterogeneous-binding sites model.

Several groups have worked on the structure of phospholipid-bound Gla domains. Gla-containing clotting factors interact in a number of distinct ways with PS head groups leading to significant membrane insertion (up to 7 Å) of the hydrophobic ω-loop exposed in the Ca^2+^ ion-bound prothrombin conformation [26–28]. Recent work involving NMR, SPR and molecular dynamic simulations suggests binding of Gla domains to PS involves one specific interaction with a single PS molecule plus other less specific interactions with Ca^2+^ ions bound to exposed phosphates [29]. In this model, these phosphate groups are provided by ‘Anything But Choline’(hence, the ‘ABC’ model). Choline has a bulky head group and sterically hinders access to the phosphate groups in the membrane that can chelate with Ca^2+^ ions associated with the Gla domains of clotting factors. Molecular dynamic simulations that deal with the membrane lipid reorganisation over long timescales that might be needed to study Gla domain–membrane insertion are difficult to perform in fine detail [30], but slower lipid reorganisations may explain the slow interactions of prothrombin with PC observed in the current study. Some previous studies have also indicated that the kinetics of ω-loop insertion might be slow and take place in both pure PC and PCPS [31], especially where the phospholipid has unsaturated acyl chains as in the current study using DOPC.

### Membrane binding and prothrombin activation

Direct evidence to implicate a weak prothrombin–phospholipid interaction as important for thrombin generation comes from studies on prothrombinase activity where prothrombin substrate delivery is separated from prothrombinase (FXa + FVa) assembly [18, 32]. Higgins et al. [18] identified a low-affinity interaction (*K*_D_ = 1 μM) between prothrombin and PCPS, which was insensitive to the phase or fluidity of the membrane. Their conclusion was that assembly of the active enzyme complex (FVa + FXa) was sensitive to membrane fluidity, but substrate, prothrombin, binding was not. Thus, prothrombin was proposed to be loosely associated (*K*_D_ ~1 μM) with the phospholipid surface in a ‘shell’ [33]. This low-affinity interaction may be optimal for substrate delivery to, and removal from, a membrane-anchored enzyme complex such as prothrombinase during the propagation phase of thrombin generation where large quantities of prothrombin need to be activated. Several studies have proposed that FX substrate and FXa product can be concentrated near the surface of a membrane by exposed PS and diffuse by ‘skating’ along the membrane surface [34, 35]. By contrast, biophysical studies by Huang et al. [28] concluded that prothrombin is embedded in the membrane and moves at the same rate as phospholipid. These authors also found a *K*_D_ value of 8 μM, for prothrombin fragment 1 binding to their model membranes and highlighted complex modes of binding suggesting heterogeneous-binding sites or multiple binding steps. Other models have also suggested that membrane fluidity and hydrophobic interactions are important in promoting correct alignment of prothrombin with Va–Xa on the membrane surface [36]; or proposed conformational changes in prothrombin resulting from membrane binding [37]. Some kinetic models also include lower *K*_D_ values for prothrombin–phospholipid interactions. [7]. In conclusion, given the importance of anionic phospholipid exposure to haemostasis, the weak, fast on/off reaction observed in the presence of DOPS is implicated in the generation of thrombin activity in vivo. The potential importance of low-affinity interactions (*K*_D_ in the μM range) should not be overlooked, especially for proteins available in relatively high concentrations, such as prothrombin, which is ~1.5 μM in plasma. A similar situation applies in the case of plasminogen, a protein involved in fibrinolysis, present in plasma at a concentration of ~2.5 μM and which binds with a *K*_D_ in the μM range to fibrin [38, 39] and cell surface receptors [40]. Whether slow, high-affinity interactions with DOPC membranes are possible in vivo is difficult to say, but this type of interaction is observed in a number of in vitro model membrane systems and should be considered when data are being analysed.

## Abbreviations

Gla: γ-Carboxyglutamic acid
SPR: Surface plasmon resonance
RU: Resonance unit
PC: Phosphatidylcholine
PS: Phosphatidylserine
DOPC: 1,2-Dioleoyl-*sn*-glycerol-3-phosphocholine
DOPS: 1,2-Dioleoyl-*sn*-glycerol-3-phospho-l-serine
DOPE: 1,2-Dioleoyl-*sn*-glycero-3-phosphoethanolamine
